# NAD salvage pathway machinery expression in normal and glaucomatous retina and optic nerve

**DOI:** 10.1186/s40478-023-01513-0

**Published:** 2023-01-22

**Authors:** James R. Tribble, Anna Hagström, Kenza Jusseaume, Emma Lardner, Raymond Ching-Bong Wong, Gustav Stålhammar, Pete A. Williams

**Affiliations:** 1 Division of Eye and Vision, Department of Clinical Neuroscience, St. Erik Eye Hospital, Karolinska Institutet, Stockholm, Sweden; 2 Centre for Eye Research Australia, Royal Victorian Eye and Ear Hospital, Melbourne, Australia; 3 Department of Surgery (Ophthalmology), The University of Melbourne, Melbourne, Australia

**Keywords:** Glaucoma, Retinal ganglion cell, Optic nerve, Neurodegeneration, Axon degeneration, NAD, Metabolism, Nicotinamide

## Abstract

Glaucoma is the leading cause of irreversible blindness and is a major health and economic burden. Current treatments do not address the neurodegenerative component of glaucoma. In animal models of glaucoma, the capacity to maintain retinal nicotinamide adenine dinucleotide (NAD) pools declines early during disease pathogenesis. Treatment with nicotinamide, an NAD precursor through the NAD salvage pathway, robustly protects against neurodegeneration in a number of glaucoma models and improves vision in existing glaucoma patients. However, it remains unknown in humans what retinal cell types are able to process nicotinamide to NAD and how these are affected in glaucoma. To address this, we utilized publicly available RNA-sequencing data (bulk, single cell, and single nucleus) and antibody labelling in highly preserved enucleated human eyes to identify expression of NAD synthesizing enzyme machinery. This identifies that the neural retina favors expression of the NAD salvage pathway, and that retinal ganglion cells are particularly enriched for these enzymes. NMNAT2, a key terminal enzyme in the salvage pathway, is predominantly expressed in retinal ganglion cell relevant layers of the retina and declines in glaucoma. These findings suggest that human retinal ganglion cells can directly utilize nicotinamide and could maintain a capacity to do so in glaucoma, showing promise for ongoing clinical trials.

**Supplementary Information:**

The online version contains supplementary material available at 10.1186/s40478-023-01513-0.

## Introduction

Age is the major risk factor for neurodegenerative disease. There is a pressing need to elucidate the underlying molecular mechanisms that drive retinal susceptibility to age. Metabolic decline may be a critical, and treatable, pathogenic component of aging and neurodegenerative disease. Neurons are highly bioenergetic and are intrinsically susceptible to mitochondrial dysfunction and metabolic failure [1]. During normal neuronal aging, there is a decrease in metabolic activity and a concomitant increase in mitochondrial stress, which, when combined with other cofactors, leads to neurodegenerative disease. Glaucoma is one of the most common neurodegenerative diseases affecting ~ 80 million patients worldwide [2]. It is characterized by the progressive dysfunction and death of retinal ganglion cells resulting in irreversible blindness. Current strategies to manage glaucoma only target a risk factor; elevated intraocular pressure, and do not address the neurodegenerative components of the disease.

**Fig. 1:**
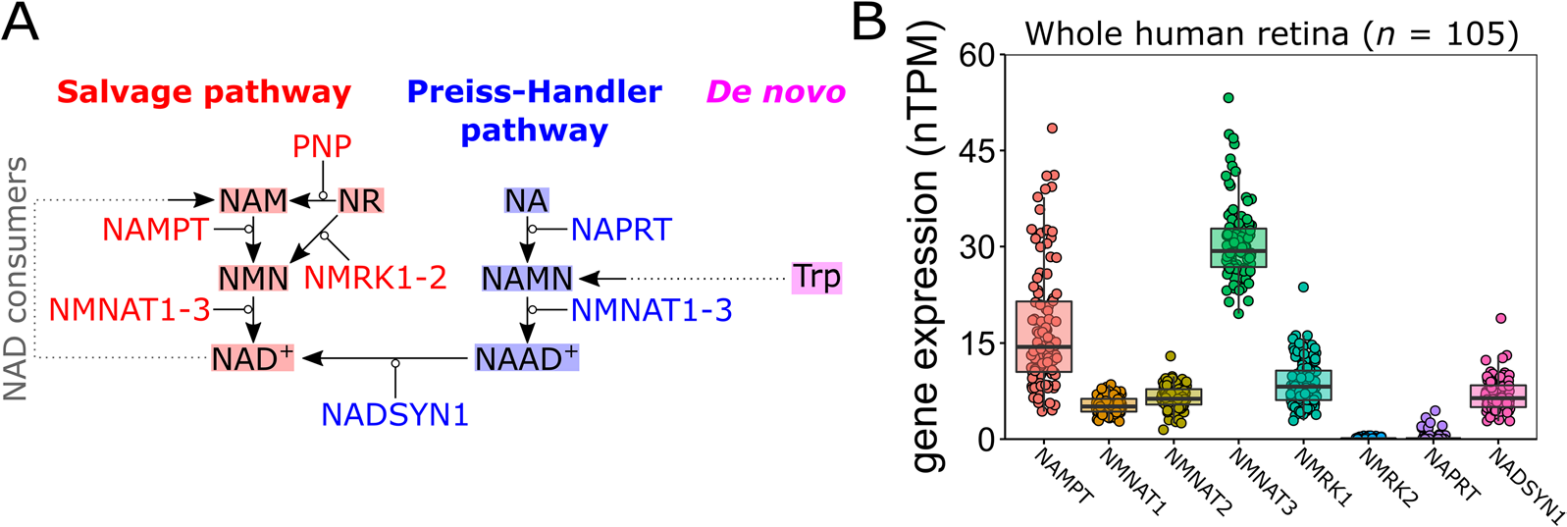
NAD synthesis pathways are represented in the retina. **A** NAD is synthesized through 3 pathways: the NAD salvage pathway, the Preiss-Handler pathway, and de novo from tryptophan. The salvage pathway (red) converts nicotinamide (NAM, a main dietary from of vitamin B_3_; also known as niacinamide) to NAD^+^ in a two-step reaction. NAM is converted to nicotinamide mononucleotide (NMN) by the enzyme NAMPT, which is then converted to NAD^+^ by NNMNATs (isoforms 1–3 are expressed in different cells and localize to different cellular compartments). Alternatively, nicotinamide riboside (NR) can be converted to either NAM (by the enzyme PNP) or NMN (by the enzyme NMRK, isoforms 1–2). NAD^+^ is recycled to NAM by NAD consumers, allowing cells to replenish NAD without constant influx of dietary precursors. The Preiss-Handler pathway (blue) converts nicotinic acid (NA; also known as niacin) to NAD^+^ in a three-step reaction. NA is converted to nicotinic acid mononucleotide (NAMN) by the enzyme NAPRT, which is converted to nicotinic acid adenine dinucleotide (NAAD^+^) by NNMNATs, and finally to NAD^+^ by the enzyme NADSYN1. Tryptophan can be converted to NAD^+^ through de novo synthesis (magenta) involving the kinurenine pathway (a six-step reaction which generates NAMN). Enzymes are shown as gene names (HGNC). **B** Gene expression of NAD-synthesizing enzymes was examined in publicly available bulk sequenced mRNA in 105 whole human retina. NAD-salvage pathway transcripts are well expressed (expect for the *NMRK2* isoform). *NAPRT* in the Preiss-Handler pathway is lowly expressed, whereas *NADSYN1* is well expressed suggesting that the retina may favor the NAD-salvage pathway

We have previously identified metabolic dysfunction and mitochondrial abnormalities occurring prior to neurodegeneration in glaucoma. This has been demonstrated both in multiple animal models of glaucoma and in glaucoma patients [3–6]. Further work by other groups has identified mitochondrial and metabolic changes in cells derived from glaucoma patients (*e.g.* Tenon’s fibroblasts [7, 8]). A major finding has been that the capacity to maintain nicotinamide adenine dinucleotide (NAD, an essential metabolite in neurons) declines in the retina in an age-dependent manner and renders retinal ganglion cells susceptible to neurodegeneration [3]. Preventing NAD depletion via administration of nicotinamide (a precursor to NAD through the NAD salvage pathway; Fig. 1) robustly prevents glaucoma in multiple animal models [3, 5, 6, 9]. Supporting a hypothesis in which age-related, pathogenically low NAD leads to glaucoma susceptibility, glaucoma patients have been demonstrated to have systemically low levels of nicotinamide (in sera) [10] and recent preliminary clinical trials have demonstrated that oral nicotinamide can improve visual function in existing glaucoma patients [11, 12]. This has initiated a large scale, multicenter randomized controlled trial (RCT) exploring nicotinamide for glaucoma prevention which is taking place in Sweden, Australia, Singapore, and the UK. Even though these preliminary studies have been successful and RCTs are now on-going, it is currently unknown if the target neurons in the retina contain or express the enzymatic machinery to utilize nicotinamide in its current form, and how this might change during glaucoma pathogenesis.

To address this, we assessed expression of NAD synthesizing enzymes in human retina in publicly available bulk, single cell, and single nucleus RNA-sequencing data. We compare this to protein expression by utilizing highly preserved enucleated eyes from St. Eriks Eye Hospital, Stockholm (*i.e.* from living donors having their eye removed due to painful glaucoma not post mortem tissue, and corresponding controls). Histological staining of high quality, tightly controlled, glaucomatous tissue from living donors identifies the enzymatic machinery in the NAD salvage pathway in the retina and optic nerve. We demonstrate that the inner retina is well-endowed with the enzymatic machinery to utilize nicotinamide directly, and identify changes in this expression profile in severe disease.

## Materials and methods

### Ethics

Access to histopathology archive samples was fully covered through biobank #366 (St Erik Eye Hospital). The study adhered to the tenets of the Declaration of Helsinki and the ethics protocols were approved by the Swedish Ethical Review Authority (2020-01525 and 2021-01036).

### Human retina

We previously utilized the Ophthalmic Pathology archive at St. Erik Eye Hospital in Stockholm, Sweden, to identify a cohort of enucleated eyes from glaucoma patients and age-matched controls [13]. Donor details are outlined in Additional file 1: Table S1. This cohort consisted of 13 control eyes and 7 glaucoma eyes. New 3 μm sections through the optic nerve head (ONH) were cut from these eyes (whole eyes embedded in paraffin wax). Sections were placed on glass slides and baked at 60 °C for 1 h. All samples labelled with the same antibody were processed as a single batch to avoid batch effects from staining. Chromogenic immunohistochemistry was performed in an automated IHC machine (Leica). Sections were deparaffinization in Bond DeWax solution, rehydrated through an ethanol gradient, prior to antigen retrieval in 1 mM EDTA buffer (pH 8.9–9.1) for 30 min at 100 °C. Sections were then washed and incubated in primary antibody for 15 min. Primary Antibodies used were: rabbit anti-NAMPT/Visfatin (Abcam, ab45890; used at 9 µg/mL), mouse anti-NMNAT1 (R&D systems, MAB5865; used at 6.7 µg/mL), mouse anti-NMNAT2 (Abcam, ab56980; used at 10 µg/mL). Slides were washed again and incubated in a polymer conjugated secondary antibody (BOND Polymer Refine Red Detection) for 50 min at room temperature followed by color development for 15 min. Two sections per eye were processed in this way with one section counterstained with hematoxylin (used only for example images to allow reference to retinal layers). Sections were dehydrated through an ethanol gradient, cleared in xylene, mounted in PERTEX, and covered with a coverslip.

### Image analysis

Slides were scanned using a Nano Zoomer s60 (Hamamatsu Photonics KK) to acquire tiled color images (40 × objective). All samples labelled with the same antibody were imaged as a single batch with imaging parameters kept constant. Semi-quantitative image analysis was performed using a protocol based on methods described previously [13]. Using NDP view2 software (Hamamatsu Photonics KK) image crops were acquired of the ONH, and the retina 2 mm (central) and 6 mm (mid-peripheral) from the center of the ONH (to the left and right). Control samples were eyes with uveal melanoma (since healthy eyes are not enucleated). In these eyes the tumor did not infringe on the central retina or optic nerve. We previously identified that the retina on the side of the tumor had significantly greater immune cell density. For the current analysis, we did not analyze the retina on the side of the tumor to avoid potential influence on metabolism. Images were exported to FIJI and deconvoluted using a vector for DAB/FastBlue/FastRed; the channel corresponding to FastRed was retained (the same chromogen as Polymer Refine Red). Retinal layers were isolated using the polygon selection tool and the mean pixel intensity was measured. The results were averaged between the left and right retina at each distance in glaucoma samples. Inner limiting membrane (ILM), nerve fiber layer (NFL), ganglion cell layer (GCL), inner plexiform layer (IPL), inner nuclear layer (INL), and the inner and outer segments of the photoreceptor layer (PS) were analyzed. Due to degenerative changes observed in glaucomatous tissue the ILM, NFL, and GCL were cropped as the ganglion cell complex (GCC), as is typically performed for in vivo clinical analysis using OCT [14], for both control and glaucoma samples when comparing these. The ONH was divided and analyzed as 5 regions of 500 µm long (in the majority of samples, there is only ~ 2–2.5 mm of ON protruding from the globe due to enucleation), beginning from a line drawn through Bruch’s membrane opening. Pixel intensity was inverted for presentation so that 0 = no signal, and 255 = maximal signal intensity.

### RNA-seq analysis of human retina

We accessed whole retina bulk RNA-sequencing data from The Genotype-Tissue Expression (GTEx) Project through The Human Protein Atlas [accessed 11/22/2022]. Data are expressed as normalized transcripts per million (nTPM) where normalization uses trimmed mean of M values to allow for between-sample comparisons. The human retina NucSeq dataset [15] was accessed through NCBI GEO (GSE135133) and imported into Seurat v4 for analysis. Using metadata provided by the authors we retained 100,055 high quality nuclei following QC filtering. Normalization was performed using the *NormalizeData()* function. Features with high cell–cell variation were identified using the *FindVariableFeatures()* function using the VST method, using default setting to return 2000 features. And linear transformation was applied to the dataset using *ScaleData()* function. Principal components were computed using the variable features and UMAP dimensional reduction was performed with the first 20 dimensions. Clustering and cell annotation ID provided by the authors were used to identify the major cell types in the retina, and unidentified subpopulations were removed from further analysis. Similarly, the human retina scRNAseq dataset [16] was available through the NCBI GEO (GSE147979) and imported into Seurat v4 for analysis. QC filtering was performed to remove low-quality cells and doublets, and the data was normalized and scaled as previously described [16]. Metadata provided by the authors were used for annotation for retinal cell types. For both NucSeq and scRNAseq, visualization of gene expression was generated using the *Dotplot()*, *Ridgeplot()* and *Vlnplot()* functions in the Seurat package.

### Statistics

Statistical analyses of antibody labelling were performed in R using an unpaired *Student’s t*-test. Unless otherwise stated, * = *P* < 0.05, ** = *P* < 0.01. For box plots, the center hinge represents the median with upper and lower hinges representing the first and third quartiles; whiskers represent 1.5 times the interquartile range.

## Results

### RNA-sequencing indicates that the neural retina favors the NAD-salvage pathway for NAD synthesis

To determine which NAD-synthesizing enzymes are present in the retina we queried publicly available RNA-sequencing data to determine which genes are transcribed. We first examined whole retina bulk RNA-sequencing data from The Genotype-Tissue Expression (GTEx) Project through The Human Protein Atlas project portal, allowing for an assessment of transcripts in human retina across a sample of 105 individuals. NAD-salvage pathway transcripts (*NAMPT*, *NMNAT1*, *NMNAT2*, *NMNAT3*) were expressed in whole retina from all individuals as was *NMRK1*, but not *NMRK2* (detected in only 32 individuals, of which all had negligible expression of < 0.5 normalized transcript per million (nTPM)). *NAMPT* and *NMNAT3* were highly variable between individuals. The Preiss-Handler pathway transcript *NAPRT* was lowly expressed and only detected in 21 individuals (< 20%) but *NADSYN1* was well expressed (Fig. 1B).

**Fig. 2:**
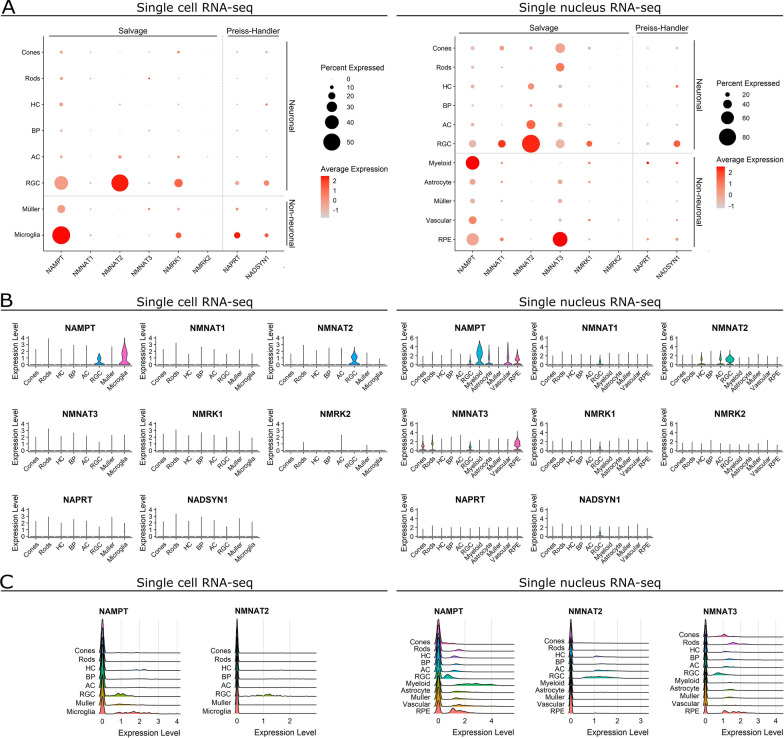
Retinal neurons predominantly express NAD-salvage pathway transcripts. **A** We examined independent datasets from single cell RNA-sequencing of normal human retina (*left*) and single nucleus RNA-sequencing of normal human retina (*right*). Expression of transcripts encoding NAD synthesizing enzyme machinery was compared across cell types of the retina. Retinal neurons demonstrate greater expression levels in a higher proportion of cells for the NAD-salvage pathway (*NAMPT*, *NMNAT1-3*) than the Preiss-Handler Pathway (*NAPRT*, *NADSYN1*). *NAMPT* expression is greater than *NMRK1-2*, suggesting a favoring of nicotinamide as an NAD-salvage pathway substrate over the alternative nicotinamide riboside. Glia and other non-neuronal support cells of the retina also favor the NAD-salvage pathway but have a greater relative expression of Preiss-Handler Pathway and *NMRK1* than neurons. **B** *NAMPT*, *NMNAT1*, and *NMNAT2* were expressed to a greater extent in RGCs over other retinal neurons. Of the non-neuronal cell types, only microglia/myeloid cells and RPE cells demonstrated strong expression of NAD synthesizing enzyme machinery. **C** For most cell types, when considering genes with high expression, the distribution of expression within cell types appeared normal, as demonstrated by ridge plots. In the single cell RNA-sequencing (*left*), distribution of *NAMPT* and *NMNAT2* suggest the possibility of distinct populations, perhaps reflecting different RGC subtypes. This was not observed in the single nucleus sequencing, where distribution of expression appeared more normal for all cell types, with the exception of *NMNAT2* in RGCs which had a greater variance, and expression of *NAMPT* and *NMNAT3* in from RPE cells. Retinal neurons: cones, rods, horizontal cells (HCs), bipolar cells (BPs), amacrine cells (ACs), and retinal ganglion cells (RGCs). Non-neuronal retinal cells: myeloid/microglia, Müller glia, astrocytes, vascular cells, and retinal pigment epithelial cells (RPE cells)

Next, we determined which cells express these transcripts by examining publicly available single cell sequencing data from human retina. We examined two independent datasets: a single cell RNA-sequencing dataset (scRNAseq) from 4 individual postmortem normal retina (~ 15,000 cells) and single nucleus RNA-sequencing dataset (NucSeq) from 4 individual postmortem normal retina (~ 100,000 nuclei in total). These allowed for identification of expression in retinal neurons (cones, rods, horizontal cells (HCs), bipolar cells (BPs), amacrine cells (ACs), retinal ganglion cells (RGCs)), and non-neuronal retinal cells (myeloid/microglia and Müller glia, and additionally astrocyte, vascular cells, and retinal pigment epithelial cells (RPE cells) in the NucSeq; Fig. 2). *NAPRT* was lowly expressed in only a small percentage of neurons in the scRNAseq and NucSeq (Fig. 2A). In contrast, *NAMPT* expression was greater in all retinal neurons (Fig. 2A) indicating a preference for the NAD-salvage pathway over the Preiss-Handler pathway. Likewise, *NADSYN1*, the terminal enzyme of the Preiss-Handler pathway, was expressed in very few retinal neurons compared to *NMNAT1-3* (Fig. 2A). *NMRK1* expression was detected in very few neurons, and *NMRK2* was almost entirely absent, indicating a preference for nicotinamide (NAM) as a substrate over nicotinamide riboside (NR) in retinal neurons (Fig. 2A). Of these, RGCs had both the highest average expression and highest percentage of cells expressing NAD-salvage pathway transcripts (Fig. 2A). *NAMPT* and *NMNAT1* appear to be particularly important in RGCs over other retinal neurons, while *NMNAT2* expression was exclusive to RGCs in the scRNAseq, with NucSeq demonstrating that other retinal neurons do express *NMNAT2,* but to a lesser extent (Fig. 2B). Examining the distribution of expression (Fig. 2C, *ridgeplots*) revealed *NAMPT* and *NMNAT1* expression to be normally distributed, whereas *NMNAT2* had a greater variance, perhaps indicating RGC-subtype specific variation in *NMNAT2*. *NMNAT3* expression was greatest in rods and cones, followed by RGCs (Fig. 2A–C), given that *NMNAT3* mRNA has a mitochondrial targeting sequence this would appear to fit with the high density of mitochondria in these neurons. However, *NMNAT3* is known to be translationally repressed due to an upstream open reading frame in the mRNA 5′UTR region and mature protein has only been identified in cells following over-expression through plasmid transfection [17]. Although rods, cones, and RGCs express *NMNAT3* it is, therefore, unlikely to have a functional role at a protein level in these cells.

In the non-neuronal cells of the retina, all transcripts were expressed in a lower percentage of cells than in neuronal cells, with the exception of *NAMPT* and *NMNAT3* (Fig. 2A–C), perhaps reflecting the lower metabolic demands of these cell types relative to neurons. *NAMPT* was expressed by a higher percentage of microglia/myeloid cells, astrocytes, Müller glia, vascular cells, and RPEs than in neuronal cell types; its average expression was greatest in microglia/myeloid cells (Fig. 2A, B). Expression of *NAPRT*, *NMRK1*, and *NADSYN1* was generally greater than in retinal neurons (except RGCs) demonstrating that non-neuronal cells in the retina may have a greater flexibility in NAD production by utilizing more substrates and pathways (Fig. 2A, B). Supporting this, when we expanded our examination to other cell types of the eye in the scRNAseq (*e.g.* corneal epithelial cells, ciliary body cells) we identified that *NMRK1* and *NADSYN1* average expression and percentage of expressing cells is far greater in the anterior chamber of the eye (Additional file 2: Fig. S1). This suggests that while the retina favors the NAD-salvage pathway, the anterior chamber favors the Preiss-Handler pathway and nicotinamide riboside as an alternative substrate to the NAD-salvage pathway.

### Antibody labelling identifies the presence of NAD salvage pathway machinery in normal retina and optic nerve

**Fig. 3:**
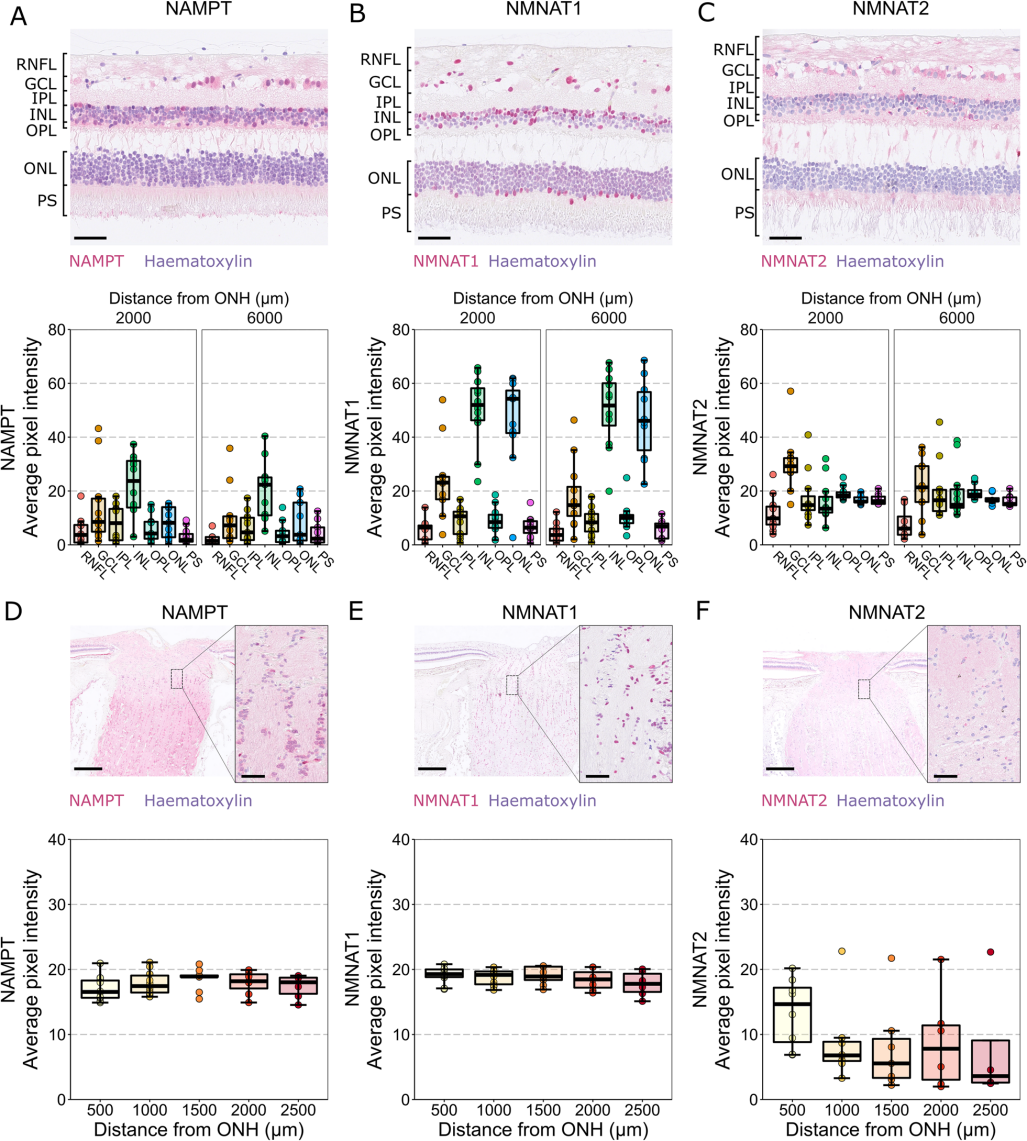
Labelling of NAD salvage pathway machinery in human retina. **A** NAMPT labelling is detected across all retinal layers, localizing to both nuclear (haematoxylin + ve) and cytoplasmic (haematoxylin −ve) cellular compartments, with the most intense labeling in the INL (*n* = 11 eyes). **B** NMNAT1 labelling is strongest in nuclear layers (GCL, INL, ONL) localizing to nuclei (*n* = 11 eyes). **C** NMNAT2 labelling localized to cytoplasmic compartments and was detected across all retinal layers, with the greatest intensity in the GCL, reflecting high expression in RGCs (*n* = 11 eyes). **D** In the ONH, NAMPT followed the same trend as in the retina, with labelling localizing to both nuclear and cytoplasmic compartments. Labeling intensity did not vary along the length of the optic nerve (*n* = 11 eyes). **E** NMNAT1 again localized only to nuclei in the ONH (glial framework), and its intensity was consistent along the length of the optic nerve (*n* = 10 eyes). **F** NMNAT2 conversely, localized only to the axonal compartments of the optic nerve and was significantly greater in the first 500 µm of the ONH (*n* = 8 eyes), potentially reflecting the density of axons relative to glial framework here, or the need for more NMNAT2 at a critical metabolic portion of the axons. N.B. these data do not allow for direct comparison of abundances of these enzymes given the nature of antibody labelling (efficiency and concentration of the antibodies etc.). *RNFL* retinal nerve fiber layer, *GCL* ganglion cell layer, *IPL* inner plexiform layer, *INL* inner nuclear layer, *OPL* outer plexiform layer, *ONL* outer nuclear layer, *PS* photoreceptor segments. Scale bars = 50 µm in **A**–**C**, 500 µm in **D**–**E** (overview images) and 50 µm in **D**–**E** (inset images)

Identification of transcript expression does not determine that a mature protein is expressed since cells employ multiple mechanisms of translational regulation. No protein data is available from the Human Protein Atlas project for any of the NAD-producing enzymes. We therefore utilized a unique resource of enucleated human eyes from the St. Erik Eye Hospital Ophthalmic Pathology archive. We previously identified and characterized 6 glaucoma and 12 control (uveal melanoma, since healthy eyes are not enucleated) eyes from this archive [13]. Importantly, since these eyes are live enucleations and fixed immediately, there are no post-mortem degenerative confounders. We used immunohistochemistry to determine localized protein expression across the retina. We focused on the NAD-salvage pathway since this was the predominant pathway identified through RNA-sequencing. NAMPT was distributed across all layers of the central and mid-peripheral retina, but was greatest in the nuclear layers, particularly the INL (*n* = 11 control eyes; Fig. 3A). NMNAT1 was clearly confined to the nuclear layers and overlapped with hematoxylin, consistent with its known nuclear localization (*n* = 11 control eyes; Fig. 3B). Conversely, NMNAT2 labelling was largely uniform across nuclear and plexiform layers and had the highest relative intensity in the RNFL compared to the rest of the retina of all the antibodies tested. NMNAT2 labelling was greatest in the GCL, this was more pronounced in the central, compared to mid-peripheral, retina where RGC density is higher (*n* = 11 control eyes; Fig. 3C).

NAMPT labelling was present in the both the axon and glial compartments of the optic nerve but was more intense in the latter; the labelling was highly uniform along the optic nerve length (*n* = 11 control eyes; Fig. 3D). NMNAT1 (*n* = 10 control eyes) and NMNAT2 (*n* = 8 control eyes) labelling demonstrated contrasted localization with NMNAT1 located only in nuclei (particularly high in glia compartments; Fig. 3E) and NMNAT2 only in axons (no labeling within or around nuclei, as can be seen in Fig. 3F *inset*). While NMNAT1 labelling was highly uniform along the optic nerve (Fig. 3E), NMNAT2 was significantly greater in the initial 500 µm (representing the pre-lamina, lamina cribrosa and initial pos-laminar region; Fig. 3F). This may reflect the increased density of axons relative to glia and connective tissue here, as well as the optic nerve head representing a particularly metabolically active portion of the optic nerve where axons first become myelinated.

### NAD salvage pathway machinery labelling is reduced in glaucoma

**Fig. 4:**
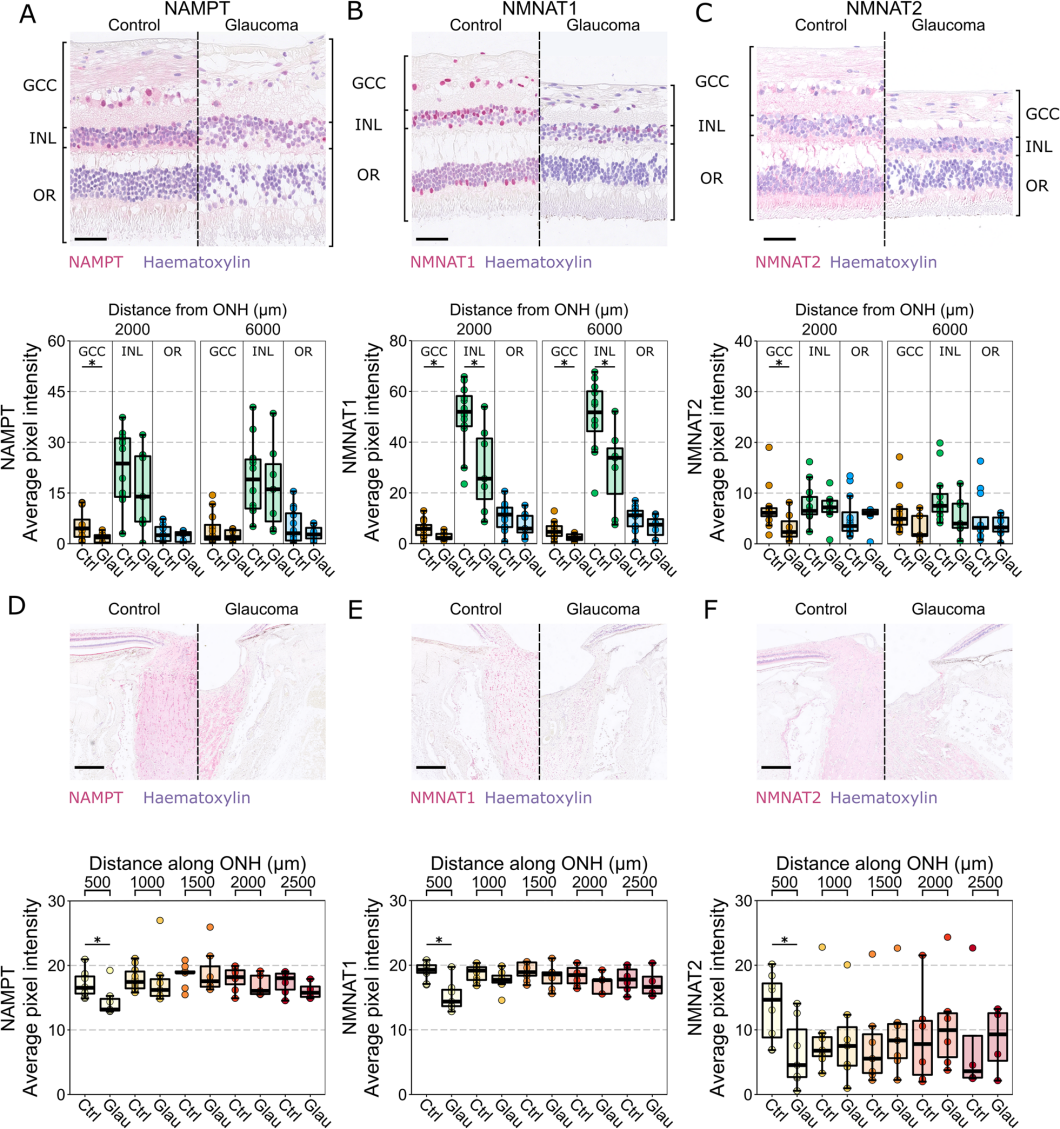
NAD salvage pathway machinery are reduced in RGC relevant layers of the retina and in the optic nerve head. **A** NAMPT labelling remains detectable across the whole retina in glaucoma but is significantly reduced in the GCC (*n* = 11 control eyes, 7 glaucoma eyes).** B** NMNAT1 is significantly reduced in both the GCC and INL in glaucoma and remains stable in the outer retina (*n* = 11 control eyes, 7 glaucoma eyes). **C** NMNAT2 is significantly reduced in the GCC in central retina and is otherwise unchanged in the INL and outer retina (*n* = 11 control eyes, 7 glaucoma eyes. **D–F** in the ONH, NAMPT, NMNAT1, and NMNAT2 labelling is significantly reduced in the first 500 µm but remain comparable to controls in the proceeding optic nerve (NAMPT: *n* = 11 control eyes, 5 glaucoma eyes; NMNAT1: *n* = 10 control eyes, 5 glaucoma eyes; NMNAT2: *n* = 8 control eyes, 5 glaucoma eyes). *GCC* ganglion cell complex, *INL* inner nuclear layer, *OR* outer retina, *ONH* optic nerve head, *Ctrl* control, *Glau* glaucoma. Scale bars = 50 µm in **A**–**C**, 500 µm in **D**–**E**

To determine if the expression patterns of these NAD salvage pathway machinery are altered in glaucoma, we compared the control eyes with 7 glaucoma eyes. Since the glaucoma tissue is from late-stage disease, we accounted for remodeling of the inner retinal layers by delineating the whole ganglion cell complex (GCC; combined RNFL, GCL, and IPL) as is common for in vivo OCT imaging studies. NAMPT labelling in the central retina was significantly reduced in the GCC in glaucomatous samples relative to controls and was not significantly altered in the INL or outer retina (*n* = 11 control eyes, 7 glaucoma eyes; Fig. 4A). NMNAT1 was significantly reduced in the GCC and INL in both central and mid-peripheral retina, with no detectable difference in the outer retina (*n* = 11 control eyes, 7 glaucoma eyes; Fig. 4B). NMNAT2 labelling was significantly reduced only in the central retina GCC in glaucomatous samples (*n* = 11 control eyes, 7 glaucoma eyes; Fig. 4C). These changes likely reflect the loss of RGC axons, cell somas, and dendrites, and potential subsequent structural and metabolic remodeling of the INL. In the ONH NAMPT was significantly reduced in the first 500 µm and was otherwise unchanged across the remaining nerve (*n* = 11 control eyes, 5 glaucoma eyes; Fig. 4D). This pattern was also evident for NMNAT1 (*n* = 10 control eyes, 5 glaucoma eyes) and NMNAT2 (*n* = 8 control eyes, 5 glaucoma eyes; Fig. 4E, F). These changes are predominantly a reflection of the loss of neural content through cupping and excavation of the ONH in this late stage of disease.

Taken together, these findings demonstrate that the inner retina and proximal optic nerve/optic nerve head is highly enriched with the machinery to directly utilize nicotinamide through the salvage pathway. However, while the ability to utilize nicotinamide remains in glaucoma, the capacity to do so may be lower during disease.

## Discussion

Vision loss from glaucoma is a significant health and economic burden. However, the only current strategies to manage glaucoma target a risk factor; elevated intraocular pressure, and do not address the neurodegenerative components of the disease. Development of neuroprotective therapies and a clear understanding of their potential mechanisms of action is of crucial importance. NAD production is an ideal target for neuroprotection in glaucoma and other age-related ophthalmic diseases. In mammals, NAD is produced through 3 primary pathways: de novo from tryptophan, the Preiss-Handler pathway from niacin, and the NAD salvage pathway from nicotinamide (and an alternate entry route in from nicotinamide riboside) [18]. The maintenance of NAD pools in neurons is most critically controlled by the NAD salvage pathway (Fig. 1). We demonstrate that the retina, as a whole, has a greater transcript abundance for the NAD salvage pathway over the Preiss-Handler pathway. Single cell/nucleus RNA sequencing data confirmed that this preference is also reflected in transcript abundance in all neuronal types of the retina. NAPRT was below expression cut-off in ~ 80% of retina in the bulk RNA-sequencing data and below expression cut-off in > 90% of all cells in the single cell and single nucleus sequencing data. These data suggest that the Preiss-Handler pathway has a very limited role in the retina. The NAD salvage pathway starts with nicotinamide and goes through a two-step reaction to become NAD. The first step to NMN is catalyzed by NAMPT, likely to be the rate limiting enzyme in this pathway. The final step of NAD production in neurons is controlled through two terminal enzymes; NMNAT1 (localized to the nucleus) and NMNAT2 (localized in the cytoplasm) [18]. This distribution is particularly evident in our retinal histology, with NMNAT1 colocalizing to hematoxylin within the retinal nuclear layers and NMNAT2 present in the cytoplasm throughout the retinal neuropil of the nuclear and plexiform layers. This is most apparent in the optic nerve where NMNAT1 is found only within glia (the only nucleated cells in the nerve) and NMNAT2 in the axonal component (RGC axons only). We have previously used the compartmentalized nature of these enzymes to identify neuroprotective mechanisms at the level of the soma (NMNAT1) [3] or axon (NMNAT2 and the WLD^S^ fusion protein) [19]. As such, both NMNAT1 and NMNAT2 may be valid treatment targets in the context of glaucoma and optic neuropathies. NAMPT was distributed across all retinal layers and localized to both nuclei and cytoplasm reflecting its need for localized NMN production at the site of both NMNAT1 and NMNAT2.

As a neuron-specific, cytosolically expressed protein, NMNAT2 is emerging as an extremely important NAD producing enzyme in axons, protecting from axon degeneration. *NMNAT2* expression is decreased in brains with Alzheimer's disease and has a highly variable expression in aged postmortem human brains [20]. We have previously demonstrated that there is down-regulation of *Nmnat2* in rodent RGCs in both an age- and disease-dependent manner [3, 21] and we hypothesized that *NMNAT2* expression levels may be a potential risk factor in human glaucoma. We expand this knowledge by demonstrating that RGCs are the predominant *NMNAT2* gene expressing neurons of the retina (reflecting the fact that they are the only retinal neuron with a true axon *i.e.* a large cytoplasmic volume), and that NMNAT2 protein is greatest in the RGC relevant layers of the retina and localizes to RGC axons in the optic nerve. NMNAT2 expression was variable in the retina and optic nerve head, which could potentially contribute to the variability of glaucoma progression. NMNAT2 has a rapid turnover time in vivo [22]. Importantly, since we used eyes that are live enucleations and fixed immediately, there are no post-mortem degenerative confounders or potential for loss of protein through rapid turnover. It is important to note, that these glaucoma eyes represent a more severe disease stage than might be first identified in the clinic and that this loss of NMNAT2 might be due to the loss of neuronal content in the retina.

There was visible variability in the labeling intensity of NMNAT1 between nuclei in the retinal nuclear layers. The single cell/nuclear sequencing data reflects this, with low percentage expression across cell types (*i.e.* expression below cut-off in the majority of cells). Co-labelling with specific cell markers would be needed to identify whether this variability reflects inter- or intra- cell type variability. It is worth noting that the expression-cut offs in the single cell/nucleus sequencing have a high threshold for detection; in the NucSeq, on average only 2266 genes per nucleus were detected (of the ~ 10,000 transcribed genes typical in a cell type). The true percentage of cells expressing these genes is likely much higher.

Non-neuronal cells in the retina and eye had greater expression of Preiss-Handler pathway genes than neuronal cells, suggesting a lower reliance on the NAD-salvage pathway. Non neuronal cells also had greater expression of *NMRK1*, suggesting a greater ability to utilize nicotinamide riboside as an alternative NAD substrate. *NAMPT* gene expression was also proportionally higher in non-neuronal cells in the retina (microglia/myeloid, astrocytes, vascular cells, and RPEs), however protein labelling appears greater in the round nuclei of the GCL and INL than in non-uniform nuclei in the IPL (typically microglia/myeloid) or in small capillary vascular walls. Conversely, there was intense labelling surrounding nuclei in the glial framework of the optic nerve head and the vasculature of the central retinal vessels (intense labelling in the RPE is also apparent in ONH overview image, Fig. 3D), which better matched the single cell/nuclear sequencing. The high expression of *NAMPT* in microglia and monocytes may reflect its importance in immune responses. *NAMPT* is a metabolic regulator of immune states, especially as a negative regulator of Sirtuins through NMN:NAD ratio [23, 24]. *NAMPT* is up-regulated in microglia in immune activation and NAMPT is secreted via exosomes into the extracellular space where is functions as both an enzyme regulating extracellular NAD, but also directly as a cytokine (*e.g.* as a toll-like receptor-4 ligand) [25]. The NAMPT inhibitor FK866, ameliorates pro-inflammatory responses in cultured microglia and in the brain following injury [26]. NAMPT was also well labelled in the axonal portion of the optic nerve matching the known role of local NMN in axonal degeneration [27]. That NAMPT labeling was largely stable relative to controls in the glaucomatous optic nerves, suggests that changes in NAMPT at least, may not be directly associated to any NMN mediated axonal degeneration.

Supplementation of high dose nicotinamide has demonstrated robust neuroprotection in glaucoma across multiple animal species and models [3, 6] and has demonstrated improvements to retinal visual function in short-term clinical trials [11, 12]. It is important to understand whether nicotinamide can be used natively within the retina, or whether metabolic changes to the molecular structure need to occur for it to be fully utilized by retinal neurons. In this study, we demonstrate that NAD salvage machinery is well-expressed in the inner retina suggesting that retinal ganglion cells have the ability to utilize nicotinamide directly. This is supported by our earlier mouse studies demonstrating that isolated retinal neurons and optic nerve cells can utilize nicotinamide directly to generate high levels of NAD [6]. Taken together, this provides strong evidence that human retinal neurons can use nicotinamide directly and gives confidence into the ongoing clinical trials. Our finding that NAMPT, NMNAT1, and NMNAT2 labelling decreases in the inner retina with severe disease might limit this NAD synthesis in more severe glaucoma patients, however, these enzymes remain detectable at 32%, 49%, and 46% respectively, supporting the ability to generate NAD even in more diseased retina. This is consistent with the known deleterious effects of complete loss of NAD production *e.g.* spontaneous knockdown of *Nmnat2* triggers Wallerian degeneration in mouse neurons [22] and reduction of function mutations in *NMNAT1* cause Leber’s congenital amaurosis [28] (a rapid photoreceptor degenerative disease resulting in blindness). Considering that glaucoma patients have reduced nicotinamide in sera [10], reduced substrate may compound this loss of NAD salvage enzymes resulting in low NAD production or a lower and less responsive dynamic range of NAD production, acting as a critical pathomechanism. These results should also aid in the design of clinical trials for other retinal diseases affecting other retinal cells (*e.g.* the high expression of NMNAT1 in photoreceptors in the outer retina).

## Conclusions

These findings demonstrate that the inner retina and optic nerve head is highly enriched with the machinery to directly utilize nicotinamide through the salvage pathway and that the ability to do so is maintained, but the capacity to do so may be lower in glaucoma.

## Supplementary Information

**Additional file 1: Table S1.** Donor details.

**Additional file 2: Fig. S1.** The single cell RNA-sequencing dataset also contains annotated cell types from other tissues of the eye. Cells in the anterior of the eye (e.g. corneal epithelial cells, ciliary body cells) had high average expression in a high percentage of cells for NMRK1, and to a lesser extent NADSYN1. This is greater in comparison to retinal cells, suggesting that while the retina favors the NAD-salvage pathway, the anterior chamber favors the Preiss-Handler pathway and nicotinamide riboside as an alternative substrate to the NAD-salvage pathway.

## Data Availability

The datasets used and/or analyzed during the current study are available from the corresponding author on reasonable request. The whole retina, bulk RNA-sequencing data was available from The Genotype-Tissue Expression (GTEx) Project accessed through The Human Protein Atlas. The human retina NucSeq dataset was accessed through NCBI GEO (GSE135133) and the scRNAseq dataset was available through the NCBI GEO (GSE147979).

## Abbreviations

GCC: Ganglion cell complex
GCL: Ganglion cell layer
ILM: Inner limiting membrane
INL: Inner nuclear layer
IPL: Inner plexiform layer
NAD: Nicotinamide adenine dinucleotide
NFL: Nerve fiber layer
NMN: Nicotinamide mononucleotide
nTPM: Normalized transcripts per million
NucSeq: Single nucleus RNA-sequencing
OCT: Optical coherence tomography
ON: Optic nerve
ONH: Optic nerve head
PS: Inner and outer segments of the photoreceptor layer
RCT: Randomized controlled trial
RGC: Retinal ganglion cell
scRNAseq: Single cell RNA-sequencing
