# Supplementary figures and images for: NAD salvage pathway machinery expression in normal and glaucomatous retina and optic nerve
Source: Acta Neuropathol Commun. 2023 Jan 22;11:18. doi: 10.1186/s40478-023-01513-0 (PMC9867855; doi:10.1186/s40478-023-01513-0)

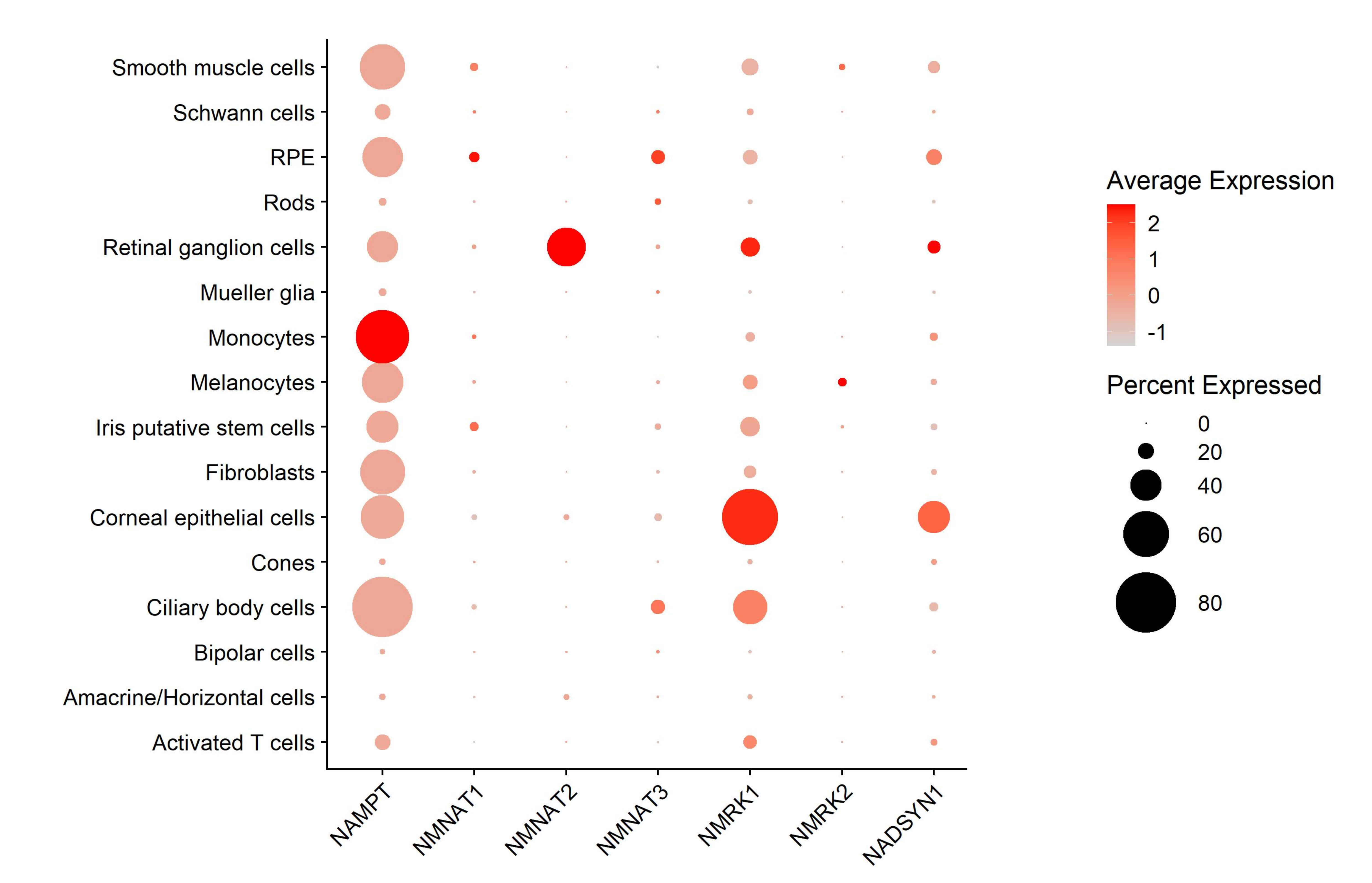

Supplement: Supplementary file 2 — Additional file 2: Fig. S1. The single cell RNA-sequencing dataset also contains annotated cell types from other tissues of the eye. Cells in the anterior of the eye (e.g. corneal epithelial cells, ciliary body cells) had high average expression in a high percentage of cells for NMRK1, and to a lesser extent NADSYN1. This is greater in comparison to retinal cells, suggesting that while the retina favors the NAD-salvage pathway, the anterior chamber favors the Preiss-Handler pathway and nicotinamide riboside as an alternative substrate to the NAD-salvage pathway. [file 40478_2023_1513_MOESM2_ESM.tif]
